# *Streptococcus pneumoniae* in Urinary Tracts of Children with Chronic Kidney Disease

**DOI:** 10.3201/eid1701.100895

**Published:** 2011-01

**Authors:** Irene Burckhardt, Stefan Zimmermann

**Affiliations:** Author affiliation: University of Heidelberg, Heidelberg, Germany

**Keywords:** Streptococcus pneumoniae, UTI, pneumococcosuria, chronic kidney disease, children, bacteria, dispatch

## Abstract

*Streptococcus pneumoniae* is not commonly considered an agent of urinary tract infections. We report 3 children with urinary tract abnormalities who had high numbers of *S. pneumoniae* in their urine (>10^4^ CFU/mL) and varying clinical symptoms.

The role of *Streptococcus pneumoniae* as an agent of septicemia, pneumonia, and meningitis is well known (*1**,**2*). However, published reports of urinary tract infection (UTI) associated with *S. pneumoniae* are scarce, and *S. pneumoniae* generally is not considered an agent of UTI in either adults or children (*3**,**4*). We report 3 children with urinary tract abnormalities and high levels of *S. pneumoniae* in their urine.

## Case Reports

In June 2008, a 23-month-old boy (case-patient 1) received care for fever and clinical signs of a UTI at the emergency department of the University of Heidelberg Children’s Hospital. He did not show signs of respiratory disease. His medical history was remarkable for bilateral cystic–dysplastic kidneys, a congenital urethral valve (surgery in October 2006), terminal kidney insufficiency and peritoneal dialysis since 2006, renal anemia, hyperparathyroidism, hyperphosphatemia, microcephalus, and failure to thrive. Blood was drawn for culture, and a urine sample was taken. After 9 hours of incubation, the blood culture was positive for *S. pneumoniae*, and the urine grew 10^5^ CFU/mL of *S. pneumoniae*. Blood leukocyte levels were elevated (20 cells/nL [normal <13 cells/nL]), as were leukocyte levels in the urine (568 cells/µL [normal <4 cells/µL]). A throat swab was negative for *S. pneumoniae*. The boy’s infection was treated successfully, and he was discharged 4 days after admission. Further analysis showed that both isolates were serotype 15B and were fully susceptible to all antibacterial drugs tested (Table 1).

**Table 1:** Characteristics of *Streptococcus pneumoniae* isolates in 3 children with chronic kidney disease, Germany, 2005–2010*

Characteristic	Case 1					Case 2			Case 3	
Sample	Blood		Urine		Urine		Urine	
Serotype	15B		15B			34			19F	
Optochin	S		S			S			S	
Bile solubility	Positive		Positive			Positive			Positive	
Oxacillin, 1 µg	S		S			S			S	
Penicillin	<0.03	S	<0.03	S		<0.03	S		0.06	S
Amoxicillin	<0.25	S	<0.25	S		<0.25	S		<0.25	S
Cefotaxime	<0.5	S	<0.5	S		<0.5	S		<0.5	S
Erythromycin	<0.06	S	<0.06	S		<0.06	S		>4.0	R
Clindamycin	0.06	S	0.06	S		0.06	S		>2	R
Tetracycline	<0.5	S	<0.5	S		<0.5	S		>8	R
Levofloxacin	1	S	1	S		<0.5	S		1.0	S
Meropenem	<0.125	S	<0.125	S		<0.125	S		<0.125	S
Vancomycin	<0.5	S	<0.5	S		<0.5	S		<0.5	S
Linezolid	<1	S	<1	S		<1	S		<1	S
Moxifloxacin	<0.25	S	<0.25	S		<0.25	S		<0.25	S
Cotrimoxazol	1/19	I	1/19	I		≤0.5/9.5	S		<0.5/9.5	S

*Values are MICs (mg/mL). S, susceptible; R, resistant; I, intermediate.

In September 2009, a 12-year-old boy (case-patient 2) sought care at the nephrology department of the University of Heidelberg Children’s Hospital for his yearly control examination 7 years after kidney transplantation. He had no clinical signs of current infection. His medical history was remarkable for kidney insufficiency, congenital obstruction and reflux in the urethral valve, kidney transplantation in 2002, ileocecal pouch, chronic transplant nephropathy, metabolic acidosis, hypertension, and renal anemia. A urine sample showed 10^5^ CFU/mL *S. pneumoniae* and 10^3^ CFU/mL *Enterobacteriaceae*. Urine leukocyte levels were slightly elevated (16 cells/µL). Further analysis showed that *S. pneumoniae* was serotype 34 and fully susceptible to all antibacterial drugs tested (Table 1).

In November 2009, a 7-year-old girl (case-patient 3) was sent to the emergency department of the University of Heidelberg Children’s Hospital by her pediatrician because of abnormal results in a control urine sample 4 weeks after percutaneous nephrolithoapraxia and concrement removal. She was known to have cystinuria and had already undergone extracorporeal shock wave treatment with concrement removal in 2004. Her temperature was slightly elevated (37.5°C), but she had no dysuria or pain. Urinalysis showed elevated leukocyte levels (158 cells/µL), and 10^4^ CFU/mL *S. pneumoniae* could be grown. Because of the mild symptoms, no antimicrobial drug treatment was started. Further analysis showed that the isolate was a 19F serotype (Table 1).

For each patient, urine was routinely cultured as follows: 2 samples of 1 µL each were placed on a 5% sheep-blood agar plate and a MacConkey agar plate. chromID CPS medium (bioMérieux, Nürtingen, Germany) was injected with 10 µL of urine. All plates were incubated for 18–24 h at 36°C ± 1°C in ambient air (*5*). Susceptibility testing was performed by using the BD Phoenix Automated Microbiology System with SMIC/ID panels (Becton Dickinson, Heidelberg, Germany).

## Discussion and Conclusions

The literature on urinary tract infections with *S. pneumoniae* is scarce. In 1980, Green and Selinger described a patient with a soft tissue abscess and a UTI caused by serotype 3 (*6*). In 2004, Dufke et al. described a patient with pyelonephritis and urosepsis caused by serotype 6A (*7*). In 1988, Nguyen and Penn determined the frequency of pneumococci in urine specimens from adults and found 38 (0.18%) of 22,744 samples positive for *S. pneumoniae* (*4*). Similarly, Miller et al. determined that the frequency of pneumococci in urine specimens from children was even lower: 43 (0.08%) of 53.499 samples (*3*). Of 28 patients, for whom clinical data were available, 5 had dysuria, and 2 had pyuria. Three asymptomatic children had medical histories of genitourinary abnormalities; 6 asymptomatic children had medical histories of recurrent UTI with *Enterobacteriaceae*. The serotypes and antibiotic susceptibilities of the respective isolates were not reported.

A UTI is defined as bacteriuria (>10^5^ CFU/mL in adults, >10^4^ CFU/mL in children) of 1 uropathogen and typical clinical signs, i.e., dysuria and urgency. Depending on the age of the patient, clinical signs might be less typical, especially in children <2 years of age. Generally pyuria is present (*8**,**9*). By contrast, asymptomatic bacteriuria is defined as a uropathogen (>10^5^ CFU/mL in adults, >10^4^ CFU/mL in children) without pyuria (<10 leukocytes/µL) (*9*).

Applying these criteria to the 3 cases in this report yields the following results. Assuming that *S. pneumoniae* is a uropathogen, case 1 is a UTI accompanied by septicemia. No other focus of infection with *S. pneumoniae* was apparent or could be identified. We believe this is an ascending UTI in a boy with known oliguria from bilateral cystic–dysplastic kidneys. Because case-patient 3 showed only mild symptoms, diagnosis of UTI is not obligatory. Nevertheless, it is not a mere pneumococcosuria because of the high numbers of leukocytes in the urine (158 cells/µL).

Case 2 is more difficult to classify. The episode described might be pneumococcosuria because the leukocyte level is not high; nevertheless, it is above normal. Reassessment of all microbiological data of case-patient 2 indicated that since 2005 we have received 29 different urine samples. In March 2007, we had already identified *S. pneumoniae* (10^4^ CFU/mL) and pyuria (92 CFU/µL). Additionally, 10^4^ CFU/mL *Proteus mirabilis* had been present and considered the cause of the pyuria; 11 of 27 samples had contained α-hemolytic streptococci >10^3^ CFU/mL (Table 2). Unfortunately, no test to differentiate between pneumococci and the other α-hemolytic streptococci was originally performed on these bacteria. Therefore, we can only speculate whether at least some of these samples contained *S. pneumoniae*. Taking these facts into account, we believe that this might be a case of bacterial persistence according to the criteria described by Chang and Shortliffe (cultures ± same organism) (*8*).

**Table 2:** Results of urine samples of 12-year-old boy with chronic kidney disease, Germany, 2005–2010

Date sample arrived in laboratory	Type of urine sample	CFU/mL	Species	Leukocyte count, cells/µL
2005 Apr	Catheter	10^5^	α-Hemolytic streptococci*	No data
		10^4^	*Proteus mirabilis*	
2005 Jul	Midstream	10^5^	α-Hemolytic streptococci	No data
		10^5^	*Proteus mirabilis*	
2005 Sep	Midstream	10^5^	*Streptococcus pyogenes*	No data
		10^3^	α-Hemolytic streptococci	
2007 Mar	Midstream	10^4^	*Streptococcus pneumoniae*	92
		10^4^	*Proteus mirabilis*	
2008 Mar	Midstream	10^4^	α-Hemolytic streptococci	54
		10^4^	*Proteus mirabilis*	
2008 Jun	Midstream	10^5^	*Proteus mirabilis*	33
		10^3^	α-Hemolytic streptococci	
2008 Jul	Midstream	10^5^	α-Hemolytic streptococci	150
2008 Nov	Midstream	10^4^	α-Hemolytic streptococci	90
2009 Mar	Midstream	10^5^	α-Hemolytic streptococci	46
2009 May	Midstream	10^5^	α-Hemolytic streptococci	24
		10^4^	*Proteus mirabilis*	
2009 Sep	Midstream	10^5^	*Streptococcus pneumoniae*	16
		10^3^	*Enterobacteriaceae*	
2010 Jan	Midstream	10^5^	α-Hemolytic streptococci	94
		10^4^	*Proteus mirabilis*	
2010 Jun	Midstream	10^5^	α-Hemolytic streptococci	124
		10^4^	*Proteus mirabilis*	

*Reference value is <4 cells/µL.

Reassessment of all available laboratory data on case-patient 1 showed that we received 3 urine samples before the described episode and 8 afterward. In none of the samples did we find *S. pneumoniae* or α-hemolytic streptococci. From case-patient 3 we did not receive any other material than described.

Further analysis of the *S. pneumoniae* isolates indicated that all 3 children were affected by different serotypes. Therefore, we do not have an indication that a single serotype has a predilection for the urinary tract. In theory, 2 cases could have been avoided by vaccination, i.e., case 3 (serotype 19F) and case 1 (serotype 15B). However, case-patient 3 was 4 years of age when the routine vaccination program aimed at children <2 years of age started in Germany in 2006. Because no catch-up program existed, this child was never vaccinated against pneumococci. Case-patient 1 was regularly vaccinated with heptavalent pneumococcal conjugate vaccine in 2006–2007 but was just 23 months old at disease onset disease, i.e., 1 month too young to be eligible for the 23-valent pneumococcal polysaccharide vaccine. Case-patient 2 is not yet vaccinated against pneumococci. Thus, we suggest that *S. pneumoniae* be added to the potential UTI-causing pathogens in children with urinary tract abnormalities.
